# PMA-qPCR to quantify viable cells in multispecies oral biofilm after disinfectant treatments

**DOI:** 10.1016/j.bioflm.2025.100281

**Published:** 2025-04-16

**Authors:** Sybille Schwendener, Manuela Flury, Joël Jenzer, Thomas Thurnheer, Lamprini Karygianni

**Affiliations:** Clinic of Conservative and Preventive Dentistry, Center for Dental Medicine, University of Zurich, Zurich, Switzerland

**Keywords:** Propidium monoazide, Viability qPCR, Universal bacterial primer, Degenerative bases, Oral biofilm, Chlorhexidine, Sodium hypochlorite, Colony forming units

## Abstract

Conventional quantitative real-time PCR (qPCR) amplifies DNA from viable and dead cells, which can lead to an overestimation of live bacteria. Viability qPCR aims to eliminate DNA from membrane-compromised cells through treatment with propidium monoazide (PMA).

Here, we evaluated PMA-qPCR to enumerate viable cells of *Actinomyces oris*, *Fusobacterium nucleatum*, *Streptococcus oralis*, *Streptococcus mutans,* and *Veillonella dispar*. Five-species oral biofilms were grown on hydroxyapatite discs for 64 h. The biofilms were exposed to 0.2 % chlorhexidine (CHX) or 3 % sodium hypochlorite (NaOCl) for 2 min, either once before cell harvest at 64 h or six times during biofilm growth. The total and single species cells were quantified by culture (CFU) and qPCR from samples with and without PMA treatment before DNA extraction. For species-specific qPCR, TaqMan assays were applied. To determine total bacteria counts, a SYBR green qPCR was established using universal degenerative primers for the conserved *dnaK* gene.

For biofilms treated once with CHX, the addition of PMA led to a 1 to 1.6 log_10_ reduction in PCR counts. This closely matched CFU and PMA-qPCR counts for total bacteria and all single species, except for *F. nucleatum*, where PMA-qPCR detected significantly more bacteria than culture. NaOCl treatment directly affected DNA and inhibited subsequent PCR amplification, even in samples without PMA. Single treatment of biofilms with 3 % NaOCl and six-fold exposure of biofilms to disinfectants resulted in no viable cell detection by culture. However, PMA did not completely prevent PCR amplification, indicating that disinfectant efficacy measured by viability PCR could be underestimated.

## 1 Introduction

In microbiology, culture-based methods are still a common technique to quantify viable bacterial cells in environmental, food, and clinical samples. Molecular techniques, especially, conventional quantitative real-time PCR (qPCR) suffer from the limitation that DNA from dead cells is amplified, leading to overestimation of live bacteria or even false-positive results. However, PCR detection is faster and superior to culture for many fastidious and non-cultivable bacterial species.

Viability qPCR methods promise to distinguish between viable and dead cells through the treatment of samples before DNA extraction with agents that can penetrate membrane-compromised cells, but not viable cells with an intact membrane. Viability qPCR methods were established with ethidium monoazide (EMA-qPCR) and propidium monoazide (PMA-qPCR) [[1], [2], [3]]. Both substances are DNA-intercalating dyes with an azide group that becomes covalently attached to DNA upon photoactivation. EMA treatment has been demonstrated to break DNA [4]. Furthermore, EMA and PMA treatments were proposed to render DNA insoluble, which results in the selective removal of DNA from dead cells during DNA extraction [1,5]. In any case, EMA and PMA treatment of samples inhibits DNA amplification from dead cells in subsequent qPCR assays, causing a clear increase in the quantification cycle (Cq) number. EMA is less selective for live cells than PMA, and it has been shown to penetrate bacterial cells with intact membranes [1,6]. While EMA treatment can underestimate viable cells, PMA uptake by dead cells may be less efficient, leading to false-positive signals in combination with PCR amplification [7]. Complete elimination of signals from membrane-compromised cells has remained a challenge in viability PCR and different strategies to optimize assays are documented [7,8]. The choice of the PCR target has been suggested to be an important experimental parameter when analyzing samples treated with viability dyes. It appears beneficial to target single-copy sequences and longer amplicons; both increase the likelihood that the target sequence in membrane-compromised cells has undergone inhibitory modification by the viability dye [7,9,10].

In oral microbiology, viability qPCR methods were applied to differentiate between viable and dead bacteria in several studies. Loozen and colleagues tested both EMA and PMA with live and heat-killed *Streptococcus mutans*, *Prevotella intermedia,* and *Aggregatibacter actinomycetemcomitans* [6]. They observed a larger signal reduction with EMA treatment but also inhibition of DNA amplification from viable cells. A significant reduction of qPCR amplification with isopropanol-killed periodontal pathogens after PMA treatment was also demonstrated with bacteria from both pure liquid culture and in vitro multispecies biofilm [11,12]. Besides in vitro studies, viable bacteria counts were determined in clinical samples using PMA-qPCR, namely in periodontitis, caries, and root canal infections [[13], [14], [15]]. Even though lower counts of bacteria were obtained when samples were pre-treated with PMA as compared to those without treatment, the completeness of dead bacteria elimination remains elusive. Alvarez and colleagues implemented the PMA-qPCR technique to determine cell mortality in a five-species oral biofilm after treatment with the antiseptic solution cetylpyridinium chloride (CPC) [16]. They estimated a 2 to 4-log reduction in cell numbers after CPC treatment, according to their PCR calibration. Viability PCR is undeniably a useful method to approach viable cell numbers in samples from different sources. However, the quantification range and accuracy are arguable, especially in complex samples and in the presence of many dead cells.

In the current study, we evaluated PMA-qPCR to detect viable cells in an in vitro oral biofilm consisting of *Actinomyces oris*, *Fusobacterium nucleatum*, *Streptococcus oralis*, *S*. *mutans,* and *Veillonella dispar*. This model was established as a supragingival biofilm in our laboratory in 2001 and has been used since then in several studies to test antimicrobial substances [[17], [18], [19], [20], [21], [22]]. The effectiveness of treatments is usually assessed by culture. The CFU of single species can be determined in this bacterial composition through distinct colony morphology. We could therefore directly compare the results from a culture with PMA-qPCR of biofilms. To assess the feasibility of molecular cell quantification in different biofilm inhibitory conditions, the five-species biofilms were grown for 64 h and treated in variable frequency with chlorhexidine (CHX) and sodium hypochlorite (NaOCl). PMA-qPCR cell counts were determined with species-specific TaqMan qPCR assays targeting single-copy or multiple 16S rDNA sequences. Furthermore, a SYBR green assay amplifying the conserved *dnaK* gene with universal degenerative primers was established to enumerate the total bacteria count. The feasibility and limits of the PMA-qPCR assays were discussed in the study.

## 2 Results

### 2.1 qPCR assay performance

Five TaqMan qPCR assays were used to quantify each unique bacterial species. They targeted species-specific DNA sequences, either single-copy gene in *S. oralis* (*rgg*) and *V. dispar* (*rpoB*) or variable regions in the multiple copies of the 16S rRNA genes (16S rDNA) in *A. oris*, *F. nucleatum,* and *S. mutans* (Table 1). The amplicon sizes ranged from 115 to 257 bp in length. Calibration curves performed with pure genomic DNA of the strains *A. oris* OMZ 745, *F. nucleatum* OMZ 598, *S. oralis* OMZ 607, *S. mutans* OMZ 918, and *V. dispar* OMZ 493 extended a linear dynamic range of at least 6 log_10_ concentrations (Supplementary Figs. S1a, b, c, d, e). PCR efficiencies were usually between 92 and 102 % in all five TaqMan qPCR assays, except for a single run with *S. mutans* and *V. dispar*, where efficiencies of 85 % and 88 % were obtained, respectively. Correlation coefficients R^2^ of the calibration curves were between 0.9871 and 0.9997. Gene copies and genome equivalent (GE) per reaction were calculated using the genome sizes and the copy number per genome indicated in Table 1. Due to the rather small number of calibration samples (total replicates 5 to 13), the limit of detection (LoD) was determined as the number of DNA copies that gave a positive qPCR signal in all (100 %) performed qPCR reactions instead of the commonly used 95 % positive detection rate for sensitivity of clinical assays [23] (Supplementary Table S1). With this approach, the LoD per reaction was estimated to be 5 GE for *S. mutans* and *S. oralis*, while GE of 29, 0.4, and 4 were determined for *A. oris*, *F. nucleatum,* and *V. dispar*, respectively (Table 1). To assess the limit of quantification (LoQ), intraassay variations of calibration replicates were calculated with the formula for the coefficient of variation (CVln) used before by others and acceptable CVln values ≤ 35 % [24,25] (Supplementary Fig. S1 for graphic representation). The estimated LoQs for the TaqMan qPCR assays were between 30 and 50 GE per reaction (Table 1).

**Table 1:** qPCR specifications: targets, oligonucleotide primers and probes, amplicon size, and limits of detection (LoD) and quantification (LoQ).

Species	Genome size/bp^a^	Target (copies/genome)	Primers and probes^b^ name and sequence (5'→3′)	Amplicon size/bp	LoD/(GE)^c^	LoQ/(GE)^c^	Reference
*Actinomyces oris*	3,184,721	16S rDNA (3x)	16S_Aori_F: TTGCTGGTTCTGGATGAGTG 16S_Aori_R: TGCAGACAGGCACAGAATATC 16S_Aori_P (probe): FAM-TGGATAACCGCATGAAAGTGTGGCT-BHQ-1	115	29	29	This study
*Fusobacterium nucleatum*	2,180,101	16S rDNA (5x)	Taq_Fnucl_F: AACTTCGATTTGGGTGGCG Taq_Fnucl_R: AGCTTTCATAATTCTAGGATGCCC Taq_Fnucl_P (probe): FAM-CCTCACAGCTAGGGACAACA-BHQ-1	129	0.4	42	[26]
*Streptococcus oralis*	1,931,548	*rgg* (1x)	rgg_Sora_F: TAGCATCTGAGCAAGAAGCAG rgg_Sora_R: GGCTCTTGAGCATTGGTTTG rgg_Sora_P (probe) FAM-AAGGTGACAGACGATGTTGCTGCT-BHQ-1	130	5	48	This study
*Streptococcus mutans*	2,032,925	16S rDNA (5x)	Taq_Smuta_F: CCAGAAAGGGACGGCTAACT Taq_Smuta_R: GCCTTTTACTCCAGACTTTCCT Taq_Smuta_P (probe) FAM-TATTGGGCGTAAAGGGAGCG-BHQ-1	122	5	46	[26]
*Veillonella dispar*	2,117,000	*rpoB* (1x)	rpoB_Vdis_F: GTAACAAAGGTGTCGTTTCTCG rpoB_Vdis_R: GCGAATAGCGTCAATTTGTYC rpoB_Vdis_P (probe): FAM-ACCCATTGGGCGTACCTTCTCGT-BHQ-1	257	4	44	This study
*Bacteria*: *A. oris, F*. *nucleatum, S. oralis, S*. *mutans, V. dispar*	–	*dnaK* (1x)	Universal primers (SYBR green Assay)dnaK-F2: CWGTTATCACAGTWCCWGCHTAC dnaK-F2Ao: ATCACCGTCCCGGCCTA dnaK-R2: ATACGTCRAAWGTWCCACCACC	183–195	425^d^	425^d^	This study

a The used genome sizes were obtained from NCBI entries: *A*. *oris* strain FDAARGOS_1051 (=DSM 23056): Nucleotide Accession No. NZ_CP066060.1; *F*. *nucleatum* strain ATCC 25586: NZ_CP028101.1; *S*. *oralis* strain ATCC 35037: NZ_LR134336.1 (GeneID of the glycoside hydrolase family 70 protein gene (*rgg)*: 49,599,429); *S*. *mutans* strain UA159 (=ATCC 700610): NC_004350.2; *V*. *dispar* strain ATCC 17748: Biosample ID SAMN00008863 (DNA-directed RNA polymerase subunit beta (*rpoB*)).

b Oligonucleotides for TaqMan or SYBR green qPCR assay. Oligonucleotide modification with fluorescein (FAM) at the 5′ end and black hole quencher-1 (BHQ-1) at the 3′ end. For primers with degenerative bases, mixed bases are indicated according to IUB code: H = A, T, C; R = G, A; W = A, T.

c Limit of detection (LoD) and limit of quantification (LoQ) are given in genome equivalent (GE) per reaction.

d The LoD and LoQ are given for *F. nucleatum*.

In addition, a SYBR green qPCR assay was established to quantify simultaneously all five bacteria with universal primers. For this approach, the core single-copy gene *dnaK* was targeted. Primers with degenerative bases were designed for segments with high sequence conservation. To reduce the degeneracy and compensate for different GC content, single mismatches between the primer and template were allowed, especially for *A. oris* which revealed an overall GC content of 67 % for the *dnaK* gene compared to *F. nucleatum*, *S. oralis*, *S. mutans,* and *V. dispar* whose GC contents were in the range of 33–43 % (Supplementary Fig. S2). Amplification of *dnaK* was evaluated for the five bacteria with 1 ng of genomic DNA at different annealing temperatures, primer pairs, and concentrations. Primers for 350-bp and 500-bp *dnaK* amplicons did not perform well in the SYBR green assay (data not shown), so we optimized the qPCR for the 200-bp fragment with the primers dnaK-F2 and dnaK-R2 (Table 1). With these primers, all species except *A. oris* showed Cq values in the range of 20–26 (Supplementary Fig. S3a). The inclusion of a short specific primer, dnaK-FAo, enabled the amplification of *A. oris* with a comparable Cq value (Fig. S3b). The smallest primer bias was observed at 56 °C (Fig. S3), which was used as annealing temperature in the SYBR green assay with 0.4 μM each dnaK-F2 and dnaK-R2 and 0.2 μM dnaK-FAo primers to amplify a 183 to 195 bp *dnaK* fragment (Table 1).

The qPCR with the universal primers showed a dynamic range of at least 5 log_10_ concentrations. Calibration curves were performed with genomic DNA of *F. nucleatum* OMZ 598 and a pool genomic DNA consisting of a mixture of *A. oris* OMZ 745, *F. nucleatum* OMZ 598, *S. oralis* OMZ 607, *S. mutans* OMZ 918 and *V. dispar* OMZ 493 in equal ratio by mass (Supplementary Figs. S1f and g). The LoD was 425 copies (equal to GE) with *F. nucleatum* DNA (Table 1) and 4178 with the pool DNA (Supplementary Table S1). The intra-assay Cq values at LoD revealed CVln values ≤ 35 % and were set equal to LoQ. For all performed SYBR green qPCR assays with *F. nucleatum* and pool DNA, the correlation coefficients R^2^ were between 0.9612 and 0.9978, and the PCR efficiency was around 58 %.

### 2.2 PMA-qPCR to distinguish between dead and viable bacteria in liquid single-species cultures

To evaluate the effect of PMA on DNA amplification from dead and viable bacterial cells, qPCR assays were performed with control samples of viable and dead bacterial cells, each sample with or without PMA treatment. Pure liquid cultures of *A. oris* OMZ 745, *F. nucleatum* OMZ 598, *S. oralis* OMZ 607, *S. mutans* OMZ 918, and *V. dispar* OMZ 493 with a density of 10^7^–10^8^ CFU/ml were used in the experiments (Table 2). Bacterial cells were killed by an incubation in 0.2 % CHX or 3 % NaOCl for 10 min. In addition, a sample of each strain was treated with 70 % isopropanol (IPA) as a control disinfectant known to cause membrane damage and applied before in viability PCR assays [1,2,11]. Killing efficiency was verified by plating. No growth or more than 5 log_10_ cell reductions were observed with all killing methods (Table 2). DNA templates were prepared from each sample, either with or without prior PMA treatment of the cells. If used, PMA was applied in a concentration of 50 μM similar to other studies where different Gram-positive and Gram-negative bacteria were analyzed [1,27]. PMA did little to affect PCR amplification from viable cells in both the species-specific TaqMan assays and the SYBR green assay with universal *dnaK* primers. The difference between Cq values (ΔCq) from viable-control samples (0.9 % NaCl) treated with and without PMA was in the range of −0.5 to 1.2 for all species except *S. mutans* (ΔCq = 3.1), indicating a low fraction of membrane-compromised and PMA-permeable cells (Table 2). For dead-cell controls, the ΔCq values of PMA-treated and untreated cells were species- and disinfectant-dependent. PMA caused an increase in ΔCq values in the range of 2–15.6 with dead cells killed by CHX and IPA treatment (Table 2). ΔCq values were larger in the SYBR green assay than in TaqMan assays. Killing of *S. mutans* through IPA treatment did only moderately inhibit PCR amplification, ΔCq values of 2.0 and 3.3 were obtained with species-specific and universal primers, respectively. Except for the IPA-killed *S. mutans* cells, IPA- and CHX treatment conferred a ΔCq > 4 for all species indicating that PMA treatment was effective according to PMA product information (https://biotium.com/wp-content/uploads/2020/06/PI-40013-40019.pdf). With NaOCl as a disinfectant, the situation was different; ΔCq was not indicative, since samples with both PMA treatment and non-PMA treatment showed increased Cq values compared with corresponding live control samples (Table 2). The NaOCl exposure seems to destroy cells extensively resulting in a visible decrease in cell pellet size after disinfection and decreased PCR amplification of the extracted DNA per se.

**Table 2:** Effect of PMA treatment on qPCR amplification from live and disinfectant-killed bacterial cells grown in pure liquid cultures.

***A.oris* OMZ 745**								
Treatment^a^	0.9 % NaCl		70 % IPA		0.2 % CHX		3 % NaOCl	
CFU/ml^b^	1.60 × 10^7^		0		4.00 × 10^1^		0	
primers	*A. oris* (16S rDNA)	universal (*dnaK*)	*A. oris* (16S rDNA)	universal (*dnaK*)	*A. oris* (16S rDNA)	universal (*dnaK*)	*A. oris* (16S rDNA)	universal (*dnaK*)
Cq – PMA^c^	23.1 (0.4)	23.9 (0.0)	23.0 (0.4)	23.6 (0.2)	23.6 (0.0)	23.7 (0.1)	39.3 (0.2)	35.9 (0.1)
Cq + PMA^c^	23.3 (0.4)	23.4 (0.0)	28.8 (0.2)	32.7 (0.3)	27.9 (0.0)	30.4 (0.1)	37.9 (0.3)	37.3 (1.4)
ΔCq^d^	0.2	−0.5	5.8	9.1	4.3	6.7	−1.4	1.4

***F. nucleatum* OMZ 598**								
Treatment^a^	0.9 % NaCl		70 % IPA		0.2 % CHX		3 % NaOCl	
CFU/ml^b^	5.27 × 10^8^		0		0		0	
primers	*F. nucleatum* (16S rDNA)	universal (*dnaK*)	*F. nucleatum* (16S rDNA)	universal (*dnaK*)	*F. nucleatum* (16S rDNA)	universal (*dnaK*)	*F. nucleatum* (16S rDNA)	universal (*dnaK*)
Cq – PMA^c^	15.7 (0.1)	19.9 (0.3)	16.2 (0.0)	20.1 (0.1)	17.6 (0.1)	24.7 (1.2)	29.3 (0.1)	39.3/ND
Cq + PMA^c^	15.9 (0.0)	21.1 (0.1)	23.0 (0.1)	34.0 (0.1	21.9 (0.0)	34.5 (0.7)	28.4 (0.1)	ND
ΔCq^d^	0.2	1.2	6.8	13.9	4.3	9.8	−0.9	ND

***S. oralis* OMZ 607**								
Treatment^a^	0.9 % NaCl		70 % IPA		0.2 % CHX		3 % NaOCl	
CFU/ml^b^	2.04 × 10^7^		2.00 × 10^1^		1.60 × 10^2^		0	
primers	*S. oralis* (*rgg*)	universal (*dnaK*)	*S. oralis* (*rgg*)	universal (*dnaK*)	*S. oralis* (*rgg*)	universal (*dnaK*)	*S. oralis* (*rgg*)	universal (*dnaK*)
Cq – PMA^c^	17.4 (0.2)	15.8 (0.2)	16.3 (0.1)	14.4 (0.1)	16.1 (0.2)	14.2 (0.0)	32.7 (0.3)	37.0 (0.3)
Cq + PMA^c^	17.6 (0.0)	16.5 (0.1)	21.4 (0.1)	24.4 (0.1)	22.9 (0.1)	29.8 (0.5)	30.1 (0.2)	35.0 (0.3)
ΔCq^d^	0.2	0.7	5.1	10	6.8	15.6	−2.6	−2

***S. mutans* OMZ 918**								
Treatment^a^	0.9 % NaCl		70 % IPA		0.2 % CHX		3 % NaOCl	
CFU/ml^b^	4.37 × 10^8^		0		0		0	
primers	*S. mutans* (16S rDNA)	universal (*dnaK*)	*S. mutans* (16S rDNA)	universal (*dnaK*)	*S. mutans* (16S rDNA)	universal (*dnaK*)	*S. mutans* (16S rDNA)	universal (*dnaK*)
Cq – PMA^c^	12.8 (0.2)	20.4 (0.1)	12.3 (0.1)	22.7 (0.0)	12.3 (0.0)	22.1 (0.2)	28.6 (0.0)	37.8/ND
Cq + PMA^c^	12.2 (0.1)	23.5 (0.1)	14.3 (0.0)	26.2 (0.3)	18.3 (0.0)	35.7 (0.4)	26.4 (0.1)	37.9 (1.0)
ΔCq^d^	0.6	3.1	2.0	3.6	6.0	13.6	−2.2	0.1

***V. dispar* OMZ 493**								
Treatment^a^	0.9 % NaCl		70 % IPA		0.2 % CHX		3 % NaOCl	
CFU/ml^b^	3.16 × 10^8^		0		0		0	
primers	*V. dispar* (*rpoB*)	universal (*dnaK*)	*V. dispar* (*rpoB*)	universal (*dnaK*)	*V. dispar* (*rpoB*)	universal (*dnaK*)	*V. dispar* (*rpoB*)	universal (*dnaK*)
Cq – PMA^c^	14.6 (0.2)	22.5 (0.1)	16.4 (0.2)	24.6 (0.5)	14.7 (0.0)	22.2 (0.0)	31.9 (0.0)	35.8 (1.9)
Cq + PMA^c^	14.9 (0.0)	22.6 (0.3)	27.2 (0.0)	36.8 (1.8)	21.5 (0.0)	32.3 (0.1)	33.5 (0.3)	38.5 (0.5)
ΔCq^d^	0.3	0.1	10.8	12.2	6.8	10.1	1.6	1.7

ND, not determined, for samples that did not pass the threshold level in qPCR reactions.

a Cells were incubated for 10 min at room temperature in 0.9 % NaCl, 70 % isopropanol (IPA), 0.2 % Chlorhexidine digluconate (CHX), and 3 % sodium hypochlorite (NaOCl).

b CFU/ml of samples was determined by plating.

c qPCR quantification cycle (Cq) values were from samples treated with 50 μM PMA (Cq + PMA) or without PMA (Cq – PMA). PCR was performed in duplicates and means, and standard deviations (in parentheses) are given.

d ΔCq is the difference in the Cq values from the PMA-treated and untreated cells: (Cq + PMA) - (Cq – PMA).

### 2.3 PMA-qPCR to enumerate viable cells in oral biofilms after disinfectant treatments

**Fig. 1:**
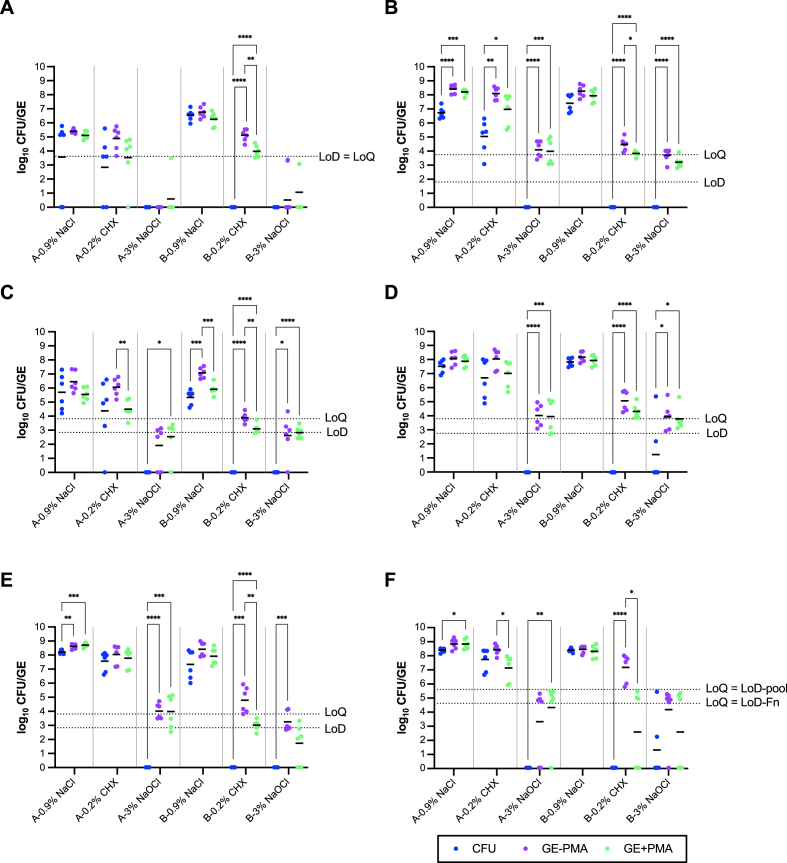
Scatter plots of log_10_ transformed bacterial counts from multispecies 64-h biofilms, representing the species (a) *A. oris* OMZ 745, (b) *F. nucleatum* OMZ 598, (c) *S. oralis* OMZ 607, (d) *S. mutans* OMZ 918, (e) *V. dispar* OMZ 493 and (f) total bacteria count. Analysis was performed by counting colony-forming units (CFU) on agar and by species-specific TaqMan qPCR (a–e) and bacteria-specific SYBR green qPCR (f) to determine genome equivalents (GE) from samples pre-treated with PMA before DNA extraction (GE+PMA) or without PMA (GE-PMA). Biofilms were exposed to 0.2 % CHX or 3 % NaOCl for 2 min once at 64 h (group A) or six-fold during biofilm growth (time points: 16 h, 20 h, 24 h, 40 h, 44 h and 48 h) (group B) as indicated below the x-axis. Control samples were treated by the same procedure with 0.9 % NaCl instead of the disinfectants. Each treatment subgroup contains data from six biological replicates with corresponding CFU, GE-PMA, and GE+PMA counts (dots). Please note that for species underrepresented in the biofilms, such as *A. oris* and *S. oralis*, CFU could not be counted with reasonable certainty in all six replicates, and data was omitted: subgroups *A. oris* A-0.9 % NaCl and A-0.2 % CHX (only 4 CFU data points); *S. oralis* A-0.2 % CHX (only 5 CFU data points). LoD and LoQ for species detection and quantification by qPCR are indicated by dotted lines, as well as means of subgroups. For total bacteria count, the LoD and LoQ for qPCR are obtained either with genomic DNA from *F. nucleatum* OMZ 598 (Fn) or a mixture of equal DNA mass of *A. oris* OMZ 745, *F. nucleatum* OMZ 598, *S. oralis* OMZ 607, *S. mutans* OMZ 918 and *V. dispar* OMZ (pool) (f). The statistical significance level is indicated with asterisks (p-values: ∗∗∗∗p < 0.0001, ∗∗∗p ≤ 0.001, ∗∗p < 0.01, ∗p < 0.05). (For interpretation of the references to colour in this figure legend, the reader is referred to the Web version of this article.)

Multispecies biofilms consisting of *A. oris* OMZ 745, *F. nucleatum* OMZ 598, *S. oralis* OMZ 607, *S. mutans* OMZ 918, and *V. dispar* OMZ 493 were grown on hydroxyapatite (HA) discs for 64 h. The biofilms were exposed to 0.2 % CHX and 3 % NaOCl, two disinfectants often used in antiseptic formulations in dental medicine. Two different disinfectant procedures were applied: either the discs with the biofilms were immersed in disinfectants for 2 min before cell harvest (time point 64 h) (treatment group A) or the biofilms were treated six-fold with the disinfectants for 2 min during biofilm growth (time points: 16 h, 20 h, 24 h, 40 h, 44 h, and 48 h) (treatment group B). The cell counts in the biofilms were quantified by culture (CFU) and qPCR (GE) with or without PMA treatment before DNA extraction (Fig. 1). Log_10_ counts were determined for 6 separate biofilms of each subgroup (treatment group A or B, disinfectant CHX or NaOCl, or control treatment with 0.9 % NaCl) and represented as single dots in Fig. 1. Variations within biological replicates in each subgroup were observed by both method culture and qPCR.

Control biofilms, treated with 0.9 % NaCl, can be assumed to contain mainly cells with intact membranes. For the single species, average ΔCq between PMA-treated and untreated samples obtained with TaqMan qPCR assays were found in the range of −0.3 to 1.7 for these control biofilms, except for *S. oralis* which had higher average ΔCq of around 3 to 4. These values corresponded to mean log_10_ GE reductions of −0.1 to 0.5 between PCR with and without PMA and a 1 log_10_ GE reduction for *S. oralis* (Fig. 1, A/B-0.9 % NaCl). The SYBR green assay with universal *dnaK* primers revealed as well only a small average ΔCq and corresponding mean log_10_ GE difference in control samples of 0–0.7 and 0.0 to 0.1, respectively (Fig. 1f, A/B 0.9 % NaCl). A good correlation between bacteria counts estimated from culture (CFU) and PMA-qPCR (GE+PMA) was observed in the control biofilms for the species *A. oris* (Fig. 1a), *S. oralis* (Fig. 1c) and *S. mutans* (Fig. 1d). Significantly fewer bacteria were detected by culturing than PMA-qPCR for the two strict anaerobes, *F. nucleatum* and *V. dispar* with log_10_ mean differences of 0.6–1.5 (Fig. 1b and e). The total bacteria count determined by culture and by SYBR green qPCR were similar in the control biofilm with a mean log_10_ difference of 0.5 to −0.1 (Fig. 1f).

A single treatment of mature biofilms with 0.2 % CHX did not kill the bacteria (Fig. 1, A-0.2 % CHX). Viable cells were detected for all species by culture. Results from PMA-qPCR correlated with CFU. Log_10_ qPCR means from samples with and without PMA differed between 1 and 1.6 (average ΔCq range 2.3–5.4), except for *V. dispar*, whose qPCR mean values were similar with and without PMA treatment (ΔLog_10_ = 0.3). For *F. nucleatum*, again, considerably lower cell counts were obtained from culture than qPCR (Fig. 1b). With the universal primers, the addition of PMA reduced Log_10_ qPCR mean by 1.6 in the single-CHX treated biofilms causing a slightly underrepresented cell count (0.6 Log_10_) compared with CFU from culture (Fig. 1f, A-0.2 % CHX).

A single treatment of biofilms with 3 % NaOCl (Fig. 1, A-3 % NaOCl) and six-fold exposure of biofilms to CHX or NaOCl (B-0.2 % CHX, B-3 % NaOCl) resulted in loss of viable cell detection by culture. An exception was only *S. mutans* that could survive the NaOCl treatment in two out of six biofilms (Fig. 1d, B-3 % NaOCl). In one biofilm, still 2.5 × 10^5^ and 2.3 × 10^5^ *S. mutans* cells were detected by CFU and PMA-qPCR after NaOCl treatment, respectively. In most samples with no colony growth on agar, PCR detection of single species and total bacteria was still possible, regardless of whether PMA was included or not. PMA treatment did, in general, reduce the qPCR signal. However, since qPCR counts without PMA were as well lowered in these harshly treated biofilms, mean log_10_ reduction by PMA addition was not pronounced, with less than 0.8 log_10_ reduction in all species except for *A. oris* in CHX-treated biofilms (Δlog_10_ = 1.1) (Fig. 1a, B-0.2 % CHX), and *V. dispar* in biofilms treated six-fold with CHX (Δlog_10_ = 1.8) and NaOCl (Δlog_10_ = 1.5) (Fig. 1e). In six-fold treated biofilms, PMA addition caused a mean log_10_ total bacteria reduction of 4.6 for CHX and 1.6 for NaOCl (Fig. 1f). Even though PCR amplification was not completely inhibited in samples with no colony detection by culture, the achieved mean qPCR counts were below the LoQ in around half of these cases in the TaqMan assays. Due to the lower sensitivity of the SYBR green assay, culture-negative samples showed mean log_10_ counts below the LoQ/LoD line (Fig. 1f).

**Fig. 2:**
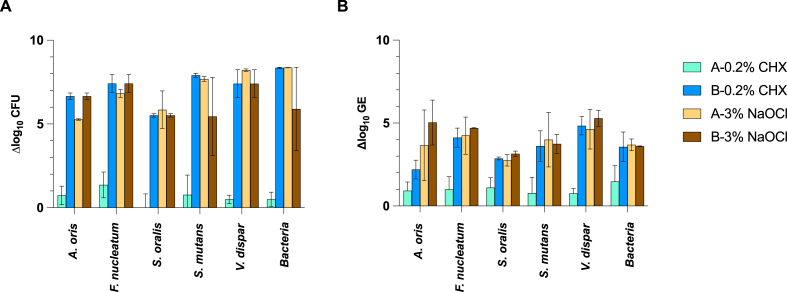
Log_10_ reduction of bacteria counts after biofilm treatment with 0.2 % CHX and 3 % NaOCl assessed by (a) culture (CFU) and (b) PMA-qPCR (GE). Reductions are expressed as the differences between the mean CFU/GE of control samples (0.9 % NaCl) and disinfectant-treated samples. The data is from Fig. 1, and key abbreviations are explained in the legend of Fig. 1. Means and standard deviations are calculated from two independent runs performed with each of three biofilms per treatment group.

Antiseptic and disinfectant efficacy is commonly tested by determining the log reduction in CFU/ml by a culture approach. According to standards, the antimicrobial intervention should reveal ≥5 log_10_ cell reductions to be considered effective. For biofilms treated with NaOCl or six-fold with CHX, >5 log_10_ reduction was obtained by culture for all species and the total bacteria count using the mean difference between control samples (NaCl) and disinfectant-treated samples (Fig. 2a). For the same samples, log_10_ reductions measured by PMA-qPCR were commonly <5 log_10_, representing the inability to completely inhibit the PCR signal from dead cells (Fig. 2b).

## 3 Methods

### 3.1 Bacterial strains and culture conditions

Experiments were performed with bacterial reference strains for in vitro supragingival biofilms, including *A*. *oris* OMZ 745, *F*. *nucleatum* OMZ 598, *S*. *oralis* OMZ 607, *S*. *mutans* OMZ 918 (UA159, ATCC 700610) and *V*. *dispar* OMZ 493 (ATCC 17748) [20,[28], [29], [30]]. All strains were cultivated either on Columbia blood agar (CBA) (Becton Dickinson and Company, Sparks, USA) containing 5 % (v/v) defibrinated sheep blood (Thermo Fisher Scientific, Waltham, USA) or in a modified fluid universal medium (mFUM) [31]. Bacterial growth for planktonic culture and biofilms was performed under anaerobic conditions (10 % H_2_, 5 % CO_2,_ and 85 % N_2_) and at a temperature of 37 °C. To grow a pure liquid culture of *V. dispar* OMZ 493, mFUM was supplemented with 1 % sodium lactate.

### 3.2 Liquid culture and killing methods

Pure cultures of all strains were grown overnight in 10 ml mFUM. Culture density was adjusted to an optical density at 660 nm (OD_660_) of 1. Per suspension, four times 1 ml was centrifuged to pellet the cells. Pellets were resuspended in a 1 ml volume of 0.9 % NaCl (control sample), 70 % isopropanol, 0.2 % CHX, and 3 % NaOCl and incubated for 10 min at room temperature. The samples were pelleted again and resuspended in 1 ml 0.9 % NaCl. Dilutions were then directly spread on agar to verify the disinfectant-killing and determine CFU (see below). Of each suspension, 350 μl without PMA and 350 μl with PMA treatment were used for DNA extraction (see below). The DNA samples were used in qPCR as viable and dead cell controls to evaluate the effect of PMA on DNA amplification.

### 3.3 Biofilms and disinfectant treatments

Multispecies biofilms consisting of *A. oris* OMZ 745, *F. nucleatum* OMZ 598, *S. oralis* OMZ 607, *S. mutans* OMZ 918, and *V. dispar* OMZ 493 were grown as previously described [28,30]. In brief, pure cultures of each bacterium were grown in mFUM for two cycles of precultures (16 and 5 h, respectively). To form the biofilm inoculum, the precultures were adjusted to an OD_660_ of 1.0 and mixed in equal volumes (1 ml each). As substrate for biofilms, sintered HA discs (Ø 9 mm; Clarkson Chromatography Products, Inc., South Williamsport, PA 17702, USA) with pre-formed pellicles were used. Therefore, HA discs were incubated in 24-well polystyrene cell culture plates with 120 μl of pasteurized whole unstimulated pooled human saliva (provided by donors of the division of Clinical Oral Microbiology and Immunology at the University of Zurich with their consent; saliva was prepared as described by Guggenheim et al. [18]) at room temperature. After 4-h incubation, the HA disks were transferred to a new 24-well plate with 1.6 ml growth medium per well (equilibrated under anaerobic conditions) consisting of 30 % mFUM and 70 % 1:2 diluted pasteurized saliva with 0.25 % NaCl. To start the 64-h biofilm (time point 0 h), 200 μl of the mixed bacterial inoculum was added to each well of the prepared HA disks in the growth medium. After 16 and 40 h, the HA disks were transferred to a new 24-well plate with 1.6 ml fresh growth medium per well, which consisted, as above, of a mixture of 30 % mFUM and 70 % diluted saliva. The carbon source in the mFUM media was 0.3 % glucose at time point 0 h, and a mixture of 0.15 % glucose and 0.15 % sucrose at time points 16 h and 40 h. Biofilms on HA disks were exposed to 0.2 % Chlorhexidine digluconate solution (CHX) (Sigma-Aldrich, St. Louis, MS, USA) or 3 % sodium hypochlorite CanalPro™ (NaOCl) (COLTENE, Altstätten, Switzerland) for 2 min at different time points. HA disks with biofilms of treatment group A were placed in 1 ml 0.2 % CHX or 3 % NaOCl at time point 64h. Disks of treatment group B were incubated in 1 ml 0.2 % CHX or 3 % NaOCl at time points 16 h, 20 h, 24 h, 40 h, 44 h and 48 h. After each disinfectant treatment and/or at the time point of medium change and before biofilm harvest (64 h), the disks were washed three times in 2 ml 0.9 % NaCl by dipping twice. For each treatment procedure, control biofilms were grown and incubated in 0.9 % NaCl instead of the disinfectant solution. Two runs of biofilm experiments were performed with 3 discs per subgroup (A-0.9 % NaCl, A-0.2 % CHX, A-3 % NaOCl, B-0.9 % NaCl, B-0.2 % CHX, and B-0.3 % NaOCl).

### 3.4 Biofilms harvest and colony plate assay

To harvest the cells from biofilms, the HA discs were vortexed for 3 min in 1 ml of 0.9 % NaCl followed by sonication of the suspension for 5 s at level 4 and 30 W (Branson Ultrasonic, Urdorf, Switzerland). Each bacterial suspension was divided into 3 fractions and used i) for PMA treatment and DNA extraction (350 μl), ii) for DNA extraction without PMA treatment (350 μl), and iii) plating to count the number of CFU. To determine the CFU, 1:10 serial dilutions of the bacterial suspension were prepared in 0.9 % NaCl ranging from 10^0^ to 10^−5^ for pure liquid culture and multispecies biofilms. Fifty μl of those dilutions were plated onto agar with a spiral diluter (EDDY JET V.1.23, IG Instrumenten-Gesellschaft, Zurich, Switzerland). After incubation of the agar plate for 3 days, colonies were counted, and species were identified through observation of colony morphology using a stereo loupe.

### 3.5 PMA treatment and DNA template preparation

PMA stock solution (1 mM in 20 % DMSO, Biotium, Inc., Fremont, CA, USA) was kept at −20 °C and protected from light. To generate PMA-qPCR templates, PMA was added at 50 μM final concentration to bacterial cell suspensions in 0.9 % NaCl. The suspensions were kept for 5 min at room temperature in the dark and then exposed to light irradiation for 2 min using the PMA-Lite™ LED Photolysis Device (Biotium). PMA-free control samples were kept for 7 min at room temperature. Both PMA-treated and untreated samples were centrifuged at 16,000 g for 5 min, the supernatant was discarded, and cell pellets were subjected to genomic DNA extraction. The GenElute™ Bacterial Genomic DNA Kit (Sigma-Aldrich) was used to prepare DNA, according to the protocol for Gram-positive bacteria with lysis buffer supplemented with 2116 × 10^6^ U/ml lysozyme and 250 U/ml mutanolysin. DNA was finally eluted from columns with 50 μl elution buffer provided by the kit. Genomic DNA used to generate qPCR calibration curves was prepared by the same procedure from pure liquid culture. The DNA concentration (mean) of the calibration samples was obtained through two measurements using the NanoDrop spectrophotometer ND-1000 (Witec AG, Littau, Switzerland).

### 3.6 Quantitative real-time PCR

The reactions were performed in a StepOnePlus Real-Time PCR System (StepOne™ Software v2.3; Applied Biosystems/Thermo Fisher Scientific). Quantification cycle (C_q_) values were obtained by automatic threshold settings. For TaqMan qPCR assays, the Luna® Universal Probe qPCR Master mix (New England Biolabs, Ipswich, MA, USA) was used with the primers and hydrolysis probes given in Table 1. DNA amplification was done in 10-μl reaction volumes using 1 μl of template DNA with 0.4 μM primers and 0.2 μM probe and the following cycling parameters: 1 min at 95 °C, followed by 45 cycles of 15 s at 95 °C and 30 s at 60 °C. For calibration curves, genomic DNA from pure cultures of *A. oris* OMZ 745, *F. nucleatum* OMZ 598, *S. oralis* OMZ 607, *S. mutans* OMZ 918, and *V. dispar* OMZ 493 was used in 10-fold dilutions ranging from 10 ng to 10^−6^ ng per reaction.

The Mastermix PowerUP SYBR Green (Thermo Fisher Scientific) was used for the SYBR green qPCR assay. Reactions were performed in 15-μl volumes using 1.5 μl of template DNA, 0.4 μM universal primers dnaK-F2 and dnaK-R2, and 0.2 μM A*. oris* primer dnaK-F2Ao (Table 1). The final qPCR cycling program included 2 min activation for UDG and polymerase at 50 °C and 95 °C, respectively, followed by 40 cycles of 15 s at 95 °C, 15 s at 56 °C, and 30 s at 72 °C. To confirm target specificity, melt curve analysis was done (ramp rate of 1.6 °C/s from 60 °C to 95 °C). Calibration curves were performed with genomic DNA from *F. nucleatum* OMZ 598 and an equal ratio by mass mixture of genomic DNA from *A. oris* OMZ 745, *F. nucleatum* OMZ 598, *S. oralis* OMZ 607, *S. mutans* OMZ 918, and *V. dispar* OMZ 493 (1:1:1:1:1). The calibration curve was obtained from measuring 10-fold dilutions ranging from 100 ng to 10^−5^ ng per reaction. DNA amplification with *dnaK*-specific primers was previously evaluated with 1 ng of genomic DNA of each single species as well as the 5-species pool at different annealing temperatures (52 °C, 54 °C, 56 °C, 58 °C, 60 °C, and 62 °C).

Genome equivalents (GE) were estimated using the formula: (g of DNA) × (6.022 × 10^23^ mol^−1^)/[(bp of genome) × (650 g mol^−1^ bp^−1^)]. The size of the genome and number of DNA copies per genome were obtained from NCBI entries and are indicated for each species in Table 1. Calibration curves were constructed by plotting the log_10_ DNA copies per reaction against their Cq values using the ggplot 2 package in R (Supplementary Figs. S1a, b, c, d, e, f, g). The obtained slope and y-intercept were used to calculate the genome equivalent in the test reactions and biofilms (multiplication factors for biofilm samples: x 50 × 2.86 (TaqMan) and x 33.3 × 2.86 (SYBR green)). Cq values were considered valid for all Cq < 40. The limit of detection (LoD) was estimated from inter-assay results from 3 or 4 independent qPCR runs with calibration standards in triplicate, duplicate, or singleton. Due to the small number of replicates (total 5 to 13), LoD was determined as the lowest copy number that gave a positive qPCR signal in all performed reactions (Supplementary Table S1). Negative control samples were taken into account: they were undetermined or gave a higher Cq than the sample used to assign the LoD. The limit of quantification (LoQ) was estimated by analyzing Cq variation at a low copy number with the formula for the coefficient of variation (CV_ln_) used before by others [24,25] and a CV_ln_ ≤ 35 % for duplicates:

$${\text{CV}}_{\ln}=\sqrt{{\left(1+E\right)}^{{\left(SD\left(Cq\right)\right)}^{2}*\;\ln\left(1+E\right)}-1}$$

where E is the qPCR efficiency: E = $10^{-\left(\frac{1}{slope}\right)}$-1

and SD (Cq) is the standard deviation of duplicated Cq values.

### 3.7 Statistical analysis

The biofilm treatment procedures A and B were conducted twice in triplicate, resulting in n = 6 samples in each subgroup, which were analyzed for cell counts by culture (CFU) and by qPCR from samples with and without PMA treatment (GE+PMA and GE-PMA). CFU could not be determined with reasonable certainty in all samples, therefore, CFU data were omitted in subgroups of species *A. oris* (A-0.9 % NaCl and A-0.2 % CHX, each only 4 CFU data points) and *S. oralis* (A-0.2 % CHX, only 5 CFU data points). CFU and GE data were log-transformed; zero in CFU and undetermined or Cq > 40 in qPCR were assigned to the value of 1 to allow for logarithmic transformation. Data were analyzed using the GraphPad Prism v.10.1.1 (GraphPad, La Jolla, CA, USA). The normal distribution was tested visually by a Normal Q-Q plot. Two-way ANOVA was used to assess the difference in bacterial cell counts for all species except for *A. oris* and *S. oralis* where a mixed-effects analysis was performed due to missing CFU values. Pairwise comparisons of the counts between CFU, GE+PMA, and GE-PMA in all subgroups were conducted using Tukey's multiple comparisons test. The significance level was set to p < 0.05.

## 4 Discussion

This study aimed to evaluate PMA-qPCR, as an alternative method to culture, to enumerate viable cells in a supragingival biofilm. For this approach, an in vitro five-species biofilm model was used, which has been established before to test antimicrobial substances and that allows viable cell determination by culture, hence direct comparison with species-specific TaqMan PMA-qPCR results [28]. Moreover, a SYBR green qPCR with universal primers targeting the *dnaK* gene was established to assess total bacteria counts by PCR in this model. To evaluate the PMA-qPCR performance with different biofilm inhibitory conditions, biofilms were treated with the disinfectants CHX (0.2 %) and NaOCl (3 %). Both substances are used in antiseptic formulations in dental medicine: CHX in antimicrobial mouthwash and NaOCl to disinfect root canals. The disinfectant treatment procedures for the 64-h biofilms, either once or six-fold for 2 min, range from bacterial growth inhibition to cell killing through expected membrane damage (CHX) or extensive cell damage (NaOCl).

Before the biofilm experiment, the effect of PMA on qPCR amplification was tested with viable and dead cells in pure liquid culture with all 5 bacterial species. The bactericidal compounds, 70 % IPA, 0.2 % CHX, and 3 % NaOCl, were applied for a 10-min incubation to kill bacteria. The addition of PMA before DNA extraction resulted in increased Cq numbers in the subsequent qPCR assays for disinfectant-killed cells but not for the corresponding viable-cell samples, which were in general little affected by the PMA treatment. The ΔCq of dead-cell controls between PMA-treated and untreated samples was in general higher in the SYBR green qPCR assay with universal primers than in the species-specific TaqMan assays. Species-specific and disinfectant-dependent differences were observed. *A. oris* and *F. nucleatum* cells were more affected by IPA treatment, while for *S. mutans* and *S. oralis,* CHX treatment caused a higher ΔCq. Especially, *S. mutans* cells seem to be little affected by IPA, judged by the low PMA-dependent inhibition of PCR amplification in both TaqMan and SYBR green assays. On the other hand, PCR inhibition by PMA was pronounced for *V. dispar* cells after IPA and CHX killing with both qPCR approaches. A reason for these differences might lie in distinct cell wall and cell membrane structures, and thereby susceptibility to potential membrane-disturbing agents and PMA uptake. IPA is known to increase membrane fluidity and cause membrane damage, and CHX is also suggested to disturb membrane integrity [32]. While clear PMA-dependent inhibition of PCR amplification was observed for IPA- and CHX-killed cells, this effect was not observed for NaOCl-killed cells. DNA extracted from NaOCl-treated samples could also, without PMA hardly be amplified by PCR. DNA might be degraded by the high oxidation capacity of NaOCl as described for NaOCl-containing disinfectants [33]. For UV light that kills *Escherichia coli*, a Cq increase in both PMA-treated and untreated cells was also reported, possibly due to DNA damage [27]. Furthermore, it was observed before that PMA can affect DNA amplification depending on the killing method. *F. nucelatum* cells killed by heat displayed more than 4 log_10_ reductions between PCR with and without PMA, while CPC-killing conferred only 2 log_10_ reductions [16]. Nocker and colleagues analyzed PMA-qPCR of bacteria killed by hypochlorite, benzalkonium chloride, and UV [27]. They suggested that inhibition of PCR correlates with the membrane damage strength of disinfectant and, therefore, uptake of PMA. Our experiments with NaOCl showed that not only the contribution to membrane damage but also to possible DNA degradation should be considered to interpret PMA-induced shift in Cq between PMA-treated and untreated dead cells.

The main goal of the study was to evaluate the viability PCR with PMA for our supragingival biofilm model: (i) with species-specific TaqMan qPCR that targeted either single-copy genes or 16S rDNA and (ii) with an SYBR green qPCR using universal primers for total bacteria counts. For control biofilms, treated with NaCl, the addition of PMA led to a good approximation of GE counts determined by qPCR and CFU counted on agar. Significantly higher PMA-qPCR counts than CFU were only observed with *F. nucleatum*, and to a smaller extent, with *V. dispar*. Both species are obligate anaerobes and might be more affected by all biofilm manipulations done under aerobe conditions. Higher PMA-qPCR counts than CFU were frequently detected with environmental samples; some authors explain this phenomenon with the presence of viable but non-culturable cells (VBNC) [34].

In our experiments, a sub-lethal treatment consisting of a 2-min incubation of 64-h biofilms in 0.2 % CHX revealed similar CFU and PMA-qPCR counts for total bacterial cells and all single species except for *F. nucleatum*, which showed significantly higher GE by PCR than CFU counts. The addition of PMA in these experiments conferred a 1 to 1.6 log_10_ reduction in GE counts compared to samples without PMA. This reduction suggests that only around 2.5–10 % of the present DNA originated from viable cells with intact membranes and indicates that qPCR without PMA could overrepresent viable cells in these samples. Moreover, in this ratio of membrane-compromised to viable cells, inhibition of PCR amplification through PMA seems to correlate well with the presence of dead cells. With exceptions of six-fold CHX-treated biofilms for *V. dispar* (Δlog_10_ = 1.8) and total bacteria (Δlog_10_ = 4.6), the mean log_10_ reductions in biofilms treated with NaOCl or six-fold with CHX showed rather smaller differences between qPCR counts from samples with and without PMA. A reason for this observation was the fact that qPCR counts without PMA were already clearly reduced. For biofilms that were treated several times with disinfectants during growth, cell proliferation can be assumed to be certainly reduced if it takes place at all. However, for 64-h biofilms only treated once before harvest with NaOCl, qPCR counts of samples without PMA could be expected to be similar to those of samples with one CHX treatment. Since this was not the case, we assume that a 2-min NaOCl incubation is already sufficient to directly affect DNA quality and inhibit subsequent PCR amplification in the biofilm experiment. In line with the results from pure liquid culture, CHX treatment of biofilms resulted in a higher difference between qPCR counts from samples with and without PMA compared with NaOCl treatment.

While PMA-qPCR and CFU counts were comparable for samples with viable cell detection on agar, this was not true for samples without CFU detection. Complete elimination of the qPCR signal from samples with no CFU detection was only observed occasionally with both qPCR approaches, e.g., in a fraction of samples analyzed by SYBR green assay and in TaqMan assays only for species that were present in lower concentrations in the biofilm, such as *A. oris* and *S. oralis* or for six-fold NaOCl-treated *V. dispar*. We estimated LoDs and LoQs for the qPCRs to evaluate the false-positive results. For species-specific analysis, one-third of the false-positive PMA-qPCR results presented a mean below the LoD and could therefore be assumed to be an unspecific background amplification. However, for all five species, at least one mean PMA-qPCR count of samples with no CFU lay above the LoD cut-off, indicating that the killing efficiency of disinfectants is difficult to interpret by PMA-qPCR results alone. The SYBR green assay with universal *dnaK* primers was less sensitive than the TaqMan assays, hence, LoD and LoQ cut-offs were higher which limited the dynamic log range for interpretation.

The log_10_ reductions caused by disinfectant treatments estimated from mean CFU were much higher than those from mean GE obtained from PMA-qPCR (Fig. 2). Treatment with NaOCl or six-fold CHX caused ≥5 log_10_ reductions for CFU, as expected for disinfectants. Due to the background amplification in PMA-qPCR, log_10_ reductions expressed as mean differences between control and treated samples were smaller than 5 log_10_. The reduction caused by six-fold CHX treatment assessed by PMA-qPCR was around 3 to 4 log_10_ for total bacteria, *S. mutans*, *S. oralis*, and *F. nucleatum*, around 2 log_10_ for *A. oris* and around 5 log_10_ for *V. dispar* in our multispecies biofilm model. Other studies that analyzed disinfectant efficiency in biofilm by viability PMA-qPCR found as well moderate log_10_ reductions with disinfectants. Alvarez and colleagues estimated 2 to 4 log_10_ reductions in oral biofilm for the species *S. oralis*, *S. gordonii*, *F. nucleatum,* and *Veillonella parvula* after treatment with CPC [16]. Treatment of 72-h oral biofilm with isopropanol for 30 min resulted in 3 log_10_ reductions for *Porphyromonas ginigvalis* and 2 log_10_ reductions for *A. actinomycetemcomitans* and *F. nucleatum* as estimated by the PMA-qPCR method [11].

Complete elimination of signals from dead cells remains a challenge in viability PCR. Optimization of PMA treatment for each species, including PMA concentration, incubation, and light exposure, could help to distinguish better between dead and live cells [7]. Since we worked with multispecies biofilms, the range of PMA optimization is limited. A more promising approach to improving PMA-qPCR reliability in mixed samples could be to use longer PCR amplicons. Longer DNA sequences might have an increased chance of being modified by PMA. Several studies have shown that false-positive results could be minimized with longer amplicons [9,10,35]. For oral bacteria, PMA-qPCR with amplicon lengths of between 200 and 400 bp was recommended [10]. Such amplicons allow satisfactory live/dead discrimination without restricting the qPCR efficiency. In our species-specific TaqMan assays, only the qPCR for *V. dispar* would meet this requirement, with an amplicon length of 257 bp. Interestingly, it was also the PMA-qPCR assay that performed best with pure *V. dispar* liquid culture and log_10_ reduction through disinfectant treatment of biofilms. Furthermore, single-copy genes were suggested as a preferred target compared to multi-copy genes for viability qPCR [7,9]. Multi-copy genes pose a higher risk that not all copies are inactivated by PMA, resulting in higher false-positive DNA amplification in qPCR. We tested both multi-copy 16S rDNA targets with *A. oris*, *F. nucleatum*, and *S. mutans* and single-copy genes with *S. oralis* and *V. dispar*. The TaqMan assays for *S. oralis* and *V. dispar* seemed to react more susceptible to PMA treatment with higher Δlog_10_ between PMA-treated and -untreated cells in biofilms and liquid culture.

Environmental and clinical biofilms consist mainly of multispecies microbial communities with undefined bacterial composition. We tested the feasibility of using universal primers with PMA-qPCR to quantify the bacteria count in our five-species biofilm model. The highly conserved 16S rDNA was not chosen as a target because of the multiple copies that, in addition, differ in number depending on the species. For example, in our setting, three 16S rDNA copies are expected for *A. oris*, four for *S. oralis* and *V. dispar,* and five copies for *F. nucleatum* and *S. mutans*. The chaperonin 60 gene (*cpn60*, also called *hsp60* and *groL*) has been used before as a universal barcode for bacteria, and degenerative primers exist that target the *hsp60* sequence [36,37]. These primers amplify a 550-bp fragment of the gene and contain up to 9 inosines in the original universal primer sequences, as well as a high degree of degeneracy [37]. Primers were later optimized by partially replacing inosines with defined degenerative bases [38]. We did not test these primers with our biofilms since the amplicon length and the level of degeneracy question a sufficient qPCR efficiency. A negative correlation between degeneracy and qPCR performance was reported before [39]. There are inconsistent results about the optimal amplicon length for viability PCR. Amplicon lengths of between 200 and 400 bp were recommended with oral bacteria [10], but 600 to 700 bp amplicons were suggested to obtain ΔCt > 10 between heat-killed and viable cells for a SYBR green assay with 16S rDNA primers targeting fish pathogens or even 1000-bp to discriminate between live and dead cells for the food pathogen *Listeria monocytogenes* [9,35].

In this study, a new SYBR green assay with degenerative primers specific for the conserved chaperonin gene *dnaK* was established. An amplicon length of around 200 bp was chosen. To reduce the degeneracy, single mismatches between primers and template were allowed except in the last 5 bases at the 3′-end. A special challenge in our setting was the amplification of the *dnaK* gene of *A. oris* which contained considerably higher GC content than the other bacteria. The dnaK-F2 and dnaK-R2 primers possessed five and three mismatches to the *A. oris* template, respectively. Since hardly any amplification was observed with this primer pair for *A. oris*, an additional species-specific primer dnaK-F2Ao was included. The primer combination dnaK-F2/F2Ao/R2 was capable of amplifying the *dnaK* gene for all single species. The qPCR assays allowed a good estimation of the cell number, as confirmed by culture. The addition of PMA caused a clear reduction of PCR signal for dead cells, as shown in the assays with single species grown in liquid culture and biofilm treated with CHX. The *dnaK* SYBR green assay presents an alternative method to quantify the total cell count in multispecies biofilm by viability PCR. The assay could in the future be extended for additional oral biofilm models or clinical samples. A disadvantage of the assay was its poor PCR efficiency, probably owing to the degeneracy and partial mismatch of the primers. Hence, the sensitivity of the *dnaK* assay is rather low, and it is not recommended for samples with DNA lower than 0.01 ng per reaction.

In conclusion, cell counts estimated from PMA-qPCR with species-specific TaqMan assays and universal dnaK primers in SYBR green assay showed a good correlation with CFU in our supragingival biofilm model as long as the fraction of viable cells was high enough to be monitored by culture. In the presence of high concentrations of dead cells, qPCR signals could not be eliminated by the addition of PMA. An unsolved issue with viability qPCR is the reliable definition of the range within PMA-qPCR counts correlate well with viable cells in a high dead cell background. The biofilm experiment with disinfectants further showed that log_10_ reduction measured with viability PCR needs to be interpreted with caution: results could be affected by the killing method per se, and disinfectant strength might be underestimated due to false-positive PCR results.

## Approval of the submitted version of the manuscript

All co-authors have read and approved the version of the manuscript that is submitted.

## CRediT authorship contribution statement

**Sybille Schwendener:** Writing – review & editing, Writing – original draft, Supervision, Methodology, Formal analysis, Data curation, Conceptualization. **Manuela Flury:** Writing – review & editing, Methodology, Investigation, Formal analysis, Data curation. **Joël Jenzer:** Methodology, Formal analysis, Conceptualization. **Thomas Thurnheer:** Writing – review & editing, Supervision, Conceptualization. **Lamprini Karygianni:** Writing – review & editing, Supervision.

## Data availability statement

Supplementary data that supports the findings of this study can be found online.

## Declaration of competing interest

The authors declare that they have no known competing financial interests or personal relationships that could have appeared to influence the work reported in this paper.
