# Supplementary material for: PMA-qPCR to quantify viable cells in multispecies oral biofilm after disinfectant treatments
Source: Biofilm. 2025 Apr 16;9:100281. doi: 10.1016/j.bioflm.2025.100281 (PMC12051517; doi:10.1016/j.bioflm.2025.100281)
Supplement: Multimedia component 1 [file mmc1.pdf]

SUPPLEMENTARY DATA

PMA-qPCR to quantify viable cells in multispecies oral biofilm after disinfectant treatments

Sybille Schwendener, Manuela Flury, Joël Jenzer, Thomas Thurnheer, Lamprini Karygianni

Clinic of Conservative and Preventive Dentistry, Center for Dental Medicine, University of Zurich, Zurich, Switzerland

SUPPLEMENTARY TABLE S1.

Detection of positive qPCR signals in reactions with low DNA concentration

Actinomyces oris OMZ 745				
Detection rate [a]	Gene copies (16S rDNA)	Genome equivalent	LoD [b] (genome copies)	LoQ [c] (genome copies)
3/11	0.9	0.3	29	29
6/11	8.7	2.9		
11/11	87	29		
Fusobacterium nucleatum OMZ 598				
Detection rate [a]	Gene copies (16S rDNA)	Genome equivalent	LoD [b]	LoQ [c]
8/8	2.1	0.4	0.4	42
8/8	21	4.2		
Streptococcus oralis OMZ 607				
Detection rate [a]	Gene copies (*rrg*)	Genome equivalent	LoD [b]	LoQ [c]
3/9	0.5	0.5	5	48
9/9	4.8	4.8		
Streptococcus mutans OMZ 918				
Detection rate [a]	Gene copies (16S rDNA)	Genome equivalent	LoD [b]	LoQ [c]
3/9	2.3	0.5	5	46
9/9	23	4.6		
Veillonella dispar OMZ 493				
Detection rate [a]	Gene copies (*rpoB*)	Genome equivalent	LoD [b]	LoQ [c]
6/8	0.4	0.4	4	44
8/8	4.4	4.4		
Bacterial pool (*A. oris* OMZ 745, *F. nucleatum* OMZ 589, *S. oralis* OMZ 607, *S. mutans* OMZ 918, *V. dispar* OMZ 493) [d]				
Detection rate [a]	Gene copies (*dnaK*)	Genome equivalent	LoD [b]	LoQ [c]
3/13	418	418	4178	4178
13/13	4178	4178		
Fusobacterium nucleatum OMZ 598				
Detection rate [a]	Gene copies (*dnaK*)	Genome equivalent	LoD [b]	LoQ [c]
0/5	43	43	425	425
5/5	425	425		

[a] Interassay results from 3 or 4 independent runs with calibration standards in duplicate or singletons.

[b] Due to the small number of samples, LoD was determined as the amount of DNA copies that gave a positive qPCR signal in all performed reactions. Negative control samples were taken into account: They were undetermined or gave a higher Cq than the sample used to assign the LoD.

[c] LoQ was obtained using the formula for the coefficient of variation (CV_{ln}) [1,2] and a $CV_{ln} \leq 35\%$ for replicates.

[d] The genomic DNA of *A. oris* OMZ 745, *F. nucleatum* OMZ 589, *S. oralis* OMZ 607, *S. mutans* OMZ 918, and *V. dispar* OMZ 493 was mixed in ratio by mass (1:1:1:1:1).

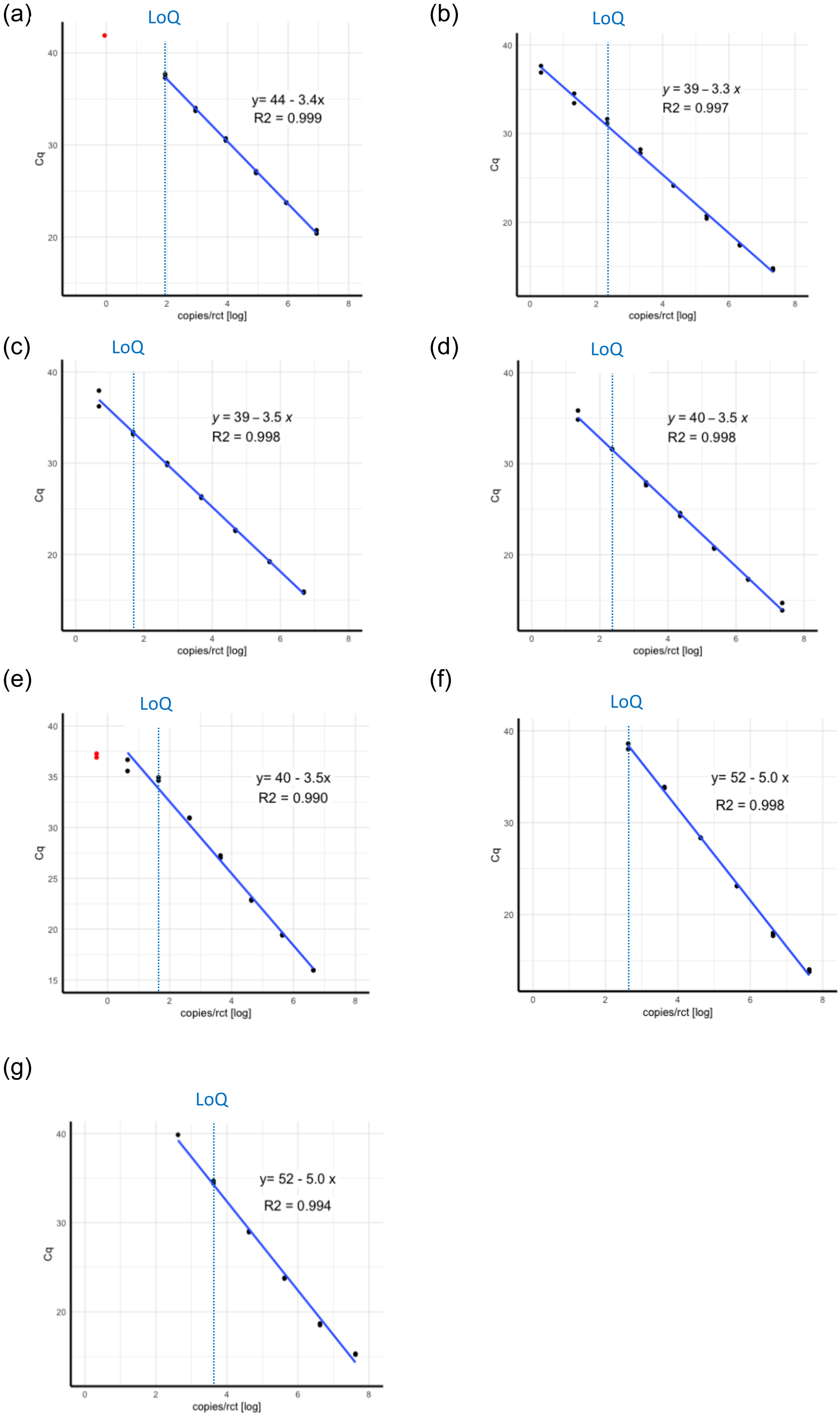

(a)
LoQ
y= 44 - 3.4x
R2 = 0.999
Cq
copies/rct [log]
(b)
LoQ
y = 39 – 3.3 x
R2 = 0.997
Cq
copies/rct [log]
(c)
LoQ
y = 39 – 3.5 x
R2 = 0.998
Cq
copies/rct [log]
(d)
LoQ
y = 40 – 3.5 x
R2 = 0.998
Cq
copies/rct [log]
(e)
LoQ
y= 40 - 3.5x
R2 = 0.990
Cq
copies/rct [log]
(f)
LoQ
y= 52 - 5.0 x
R2 = 0.998
Cq
copies/rct [log]
(g)
LoQ
y= 52 - 5.0 x
R2 = 0.994
Cq
copies/rct [log]

SUPPLEMENTARY FIG. S1. Calibration curves for quantitative real-time PCR (a-e) with species-specific primers in TaqMan assay or (f, g) universal primers in SYBR green assay. The genomic template DNA used was from (a) *Actinomyces oris* OMZ 745, (b) *Fusobacterium nucleatum* OMZ 598, (c) *Streptococcus oralis* OMZ 607, (d) *Streptococcus mutans* OMZ 918, (e) *Veillonella dispar* OMZ 493 and (f) *Fusobacterium nucleatum* OMZ 598, and (g) a mixture of genomic DNA from *A. oris* OMZ 745, *F. nucleatum* OMZ 598, *S. oralis* OMZ 607, *S. mutans* OMZ 918 and *V. dispar* OMZ 493 in ratio by mass (1:1:1:1:1). The DNA copies per reaction (log_{10}) and the Cq values for 10-fold diluted samples are shown. Cq values excluded from calculations are shown in red. The linear regression equation, the regression coefficient R^2, and the limit of quantification (LoQ) are indicated.

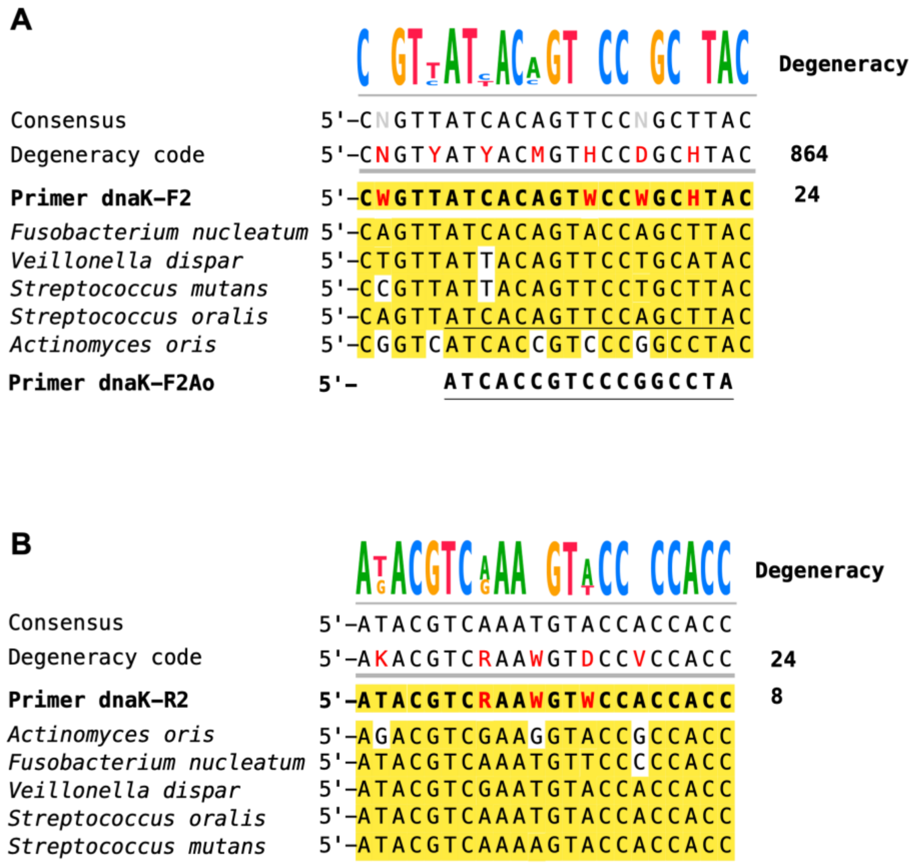

SUPPLEMENTARY FIG. S2. The dnaK-F2 and dnaK-F2Ao (A) and dnaK-R2 (B) primer sequences used for the amplification of the conserved bacterial *dnaK* gene in the SYBR green qPCR assay. The consensus sequence and degeneracy code are given for the alignment of the *Actinomyces oris*, *Fusobacterium nucleatum*, *Streptococcus oris*, *Streptococcus mutans,* and *Veillonella dispar* sequences. Mismatches between the bacterial sequences and the primers are indicated by missing yellow highlights. The alignments were exported from SnapGen Viewer software and edited manually. The degeneracy of primers is defined as the number of oligonucleotides comprising the primer due to mixed bases inclusion during DNA synthesis. IUB code for mixed bases: K = G, T; V = G, A, C; M = A, C; D = G, A, T; W = A, T; N = G, A, T, C; R = G, A; H = A, T, C; and Y = T, C.

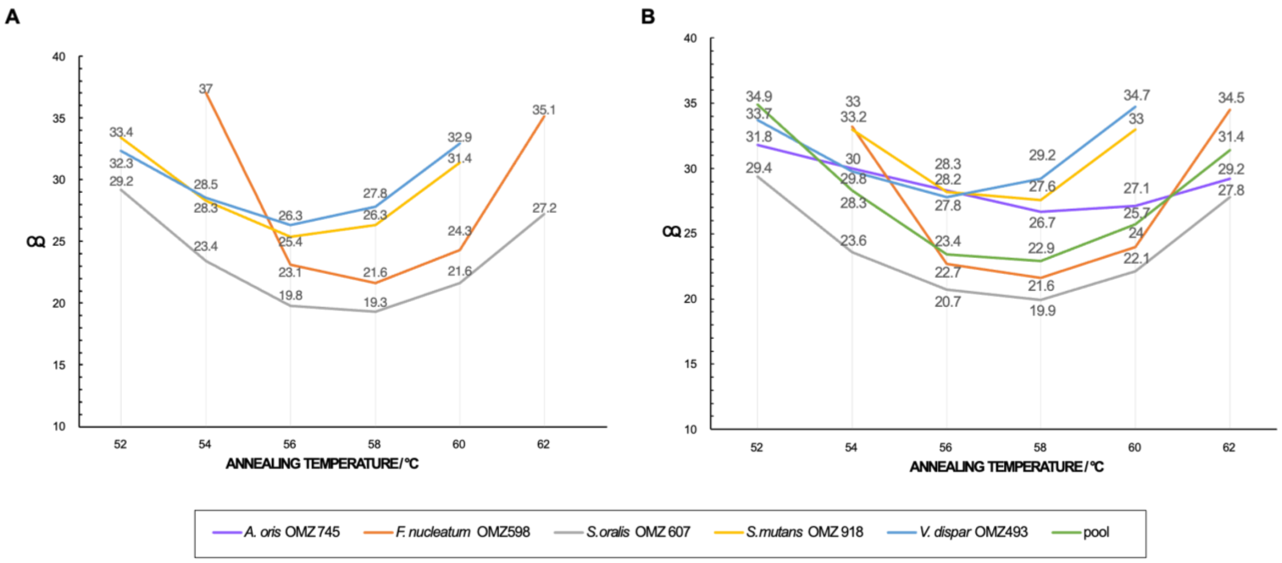

SUPPLEMENTARY FIG. S3. The quantification cycle (Cq) is represented as a function of annealing temperature for the *dnaK* SYBR green qPCR assay (a) with 0.4 µM primers dnaK-F2 and dnaK-R2 and (b) with 0.4 µM primers dnaK-F2 and dnaK-R2 and 0.2 µM primer dnaK-F2Ao. Templates are indicated and consisting of 1 ng genomic DNA from *Actinomyces oris* OMZ 745, *Fusobacterium nucleatum* OMZ 598, *Streptococcus oralis* OMZ 607, *Streptococcus mutans* OMZ 918, *Veillonella dispar* OMZ 493 and a mixture of genomic DNA from all five species in equal ratio by mass (pool) (only in b).

REFERENCES

[1] Forootan A, Sjoback R, Bjorkman J, Sjogreen B, Linz L, Kubista M. Methods to determine limit of detection and limit of quantification in quantitative real-time PCR (qPCR). Biomol Detect Quantif 2017, Jun;12:1–6. https://doi.org/10.1016/j.bdq.2017.04.001.

[2] Klymus K.E, Ruiz Ramos D.V, Thompson N.L, Richter C.A. Development and testing of species-specific quantitative PCR assays for environmental DANN applications. J Vis Exp 2020, Nov 5;165. https://doi.org/10.3791/61825.
